# Welfare Resilience at the Onset of the COVID‐19 Pandemic in a Selection of European Countries: Impact on Public Finance and Household Incomes

**DOI:** 10.1111/roiw.12530

**Published:** 2021-07-01

**Authors:** Olga Cantó, Francesco Figari, Carlo V. Fiorio, Sarah Kuypers, Sarah Marchal, Marina Romaguera‐de‐la‐Cruz, Iva V. Tasseva, Gerlinde Verbist

**Affiliations:** ^1^ Universidad de Alcalá and EQUALITAS; ^2^ University of Insubria ISER University of Essex CeRP Collegio Carlo Alberto and Dondena Bocconi University; ^3^ University of Milan Irvapp‐FBK and Dondena Bocconi University; ^4^ Herman Deleeck Centre for Social Policy University of Antwerp; ^5^ Universidad Antonio de Nebrija; ^6^ Department of Social Policy London School of Economics and Political Science

**Keywords:** COVID‐19, household incomes, tax‐benefit microsimulation, income protection, cross‐country comparison

## Abstract

This paper assesses the impact on household incomes of the COVID‐19 pandemic and governments’ policy responses in April 2020 in four large and severely hit EU countries: Belgium, Italy, Spain and the UK. We provide comparative evidence on the level of relative and absolute welfare resilience at the onset of the pandemic, by creating counterfactual scenarios using the European tax‐benefit model EUROMOD combined with COVID‐19‐related household surveys and timely labor market data. We find that income poverty increased in all countries due to the pandemic while inequality remained broadly the same. Differences in the impact of policies across countries arose from four main sources: the asymmetric dimension of the shock by country, the different protection offered by each tax‐benefit system, the diverse design of discretionary measures and differences in the household level circumstances and living arrangements of individuals at risk of income loss in each country.

## 1 Introduction

The COVID‐19 pandemic has led to a worldwide economic downturn worse than the one that characterized the 2008 Great Recession. The potential impact on GDP, although mostly unpredictable still today without a clear knowledge of the further development of the health emergency, can lead to a massive slump in economic development (Dorn *et al*., 2020). OECD estimates for the initial direct impact of the crisis revealed a decline in annual GDP growth of around 2 percentage points for each month of economic shutdown (OECD, 2020a). Focusing on the situations faced by workers, the International Labour Organization estimated initially a rise in global unemployment of between 3 percent and 13 percent, with underemployment expected to increase on a large scale and the decline in economic activity and travel limits impacting both manufacturing and services (ILO, 2020).

To limit the spread of the virus governments across Europe restricted or completely shut down non‐essential economic activities. These containment measures resulted in unprecedented demand and supply shocks. Between the first and second quarter of 2020, GDP fell drastically in some European countries—by 11.8 percent in Belgium, 13 percent in Italy, 17.9 percent in Spain and 18.8 percent in the UK, making them some of the worst affected countries economically in Europe.^1^

The picture described above, as well as the lessons of previous recessions such as the one of 2008, suggest that the downturn due to the COVID‐19 pandemic will overshadow European economies for years to come, through a legacy of unemployment, public debt and long‐lasting impacts on household incomes (Jenkins *et al*., 2013). As Saez and Zucman (2020, p. 1) rightly point out, governments “can prevent a very sharp but short recession from becoming a long‐lasting depression” by acting as payer of last resort: providing insurance to the affected workers and making sure that cash flows to idle workers and businesses immediately. Governments across Europe indeed swiftly reacted to the economic impact of the COVID‐19 shock by adjusting existing welfare policies or introducing new emergency measures. These policies were often innovative and introduced at a much larger scale compared to the policy responses to the 2008 Great Recession (Moreira and Hick, 2021).

In light of these policy responses, this paper assesses the welfare resilience of household incomes during the pandemic in a cross‐country perspective. In particular, we analyze the extent to which the tax‐benefit systems of four large and severely hit European countries—Belgium, Italy, Spain and the UK—provided income stabilization for those who lost all or part of their earnings as a consequence of the pandemic and restrictions to economic activities.

The countries studied in this paper have experienced high levels of infection rates and many deaths in their populations. Italy was the European country that experienced the first sudden outbreak at the end of February 2020. Subsequently, within the first half of March 2020 both Spain and Belgium started to follow a rapid increase in infections and deaths and by the end of March 2020 that was also happening in the UK. At the end of 2020, the four countries registered some of the highest number of deaths per million inhabitants in Europe (OECD/European Union, 2020).

We focus on the impact in the first month of the COVID‐19 pandemic (April 2020) as the lockdown was in most countries the strictest at that time. Focussing on a single month has some clear advantages for measuring welfare resilience to an unexpected shock. First, it is easier to identify who was affected by the earnings and employment shocks in each country as this is directly linked to the national rules of the economic shutdown and to each country’s labor market structure. Second, it allows for an evaluation of both country‐specific short‐term tax‐benefit automatic stabilizers and emergency policy measures before any of the EU‐level initiatives to cushion the shock kicked in.

We measure the amount of income insurance that individuals and their households received from the welfare state in April 2020, effectively providing a measure of the resilience of welfare systems at the beginning of the crisis. A comparative perspective across the most severely hit countries is warranted as cross‐country differences in welfare resilience may be considerable. Indeed, Dolls *et al*. (2012) show that automatic stabilizers differ greatly across countries, particularly in the case of asymmetric shocks. Moreover, in the European context there exists an important variation in income stabilization mechanisms of taxes and benefits, which in some countries, especially in Southern Europe, are poorly designed to face times of emergency. Besides automatic stabilizers, the emergency policy measures introduced by many European governments to support the most vulnerable (OECD, 2020b) also differ across countries, but the efficacy of these policies has still to be assessed empirically.

At the time of writing, the possibilities for empirical analysis are constrained by the lack of up‐to‐date and longitudinal information on household net incomes, which usually only becomes available a few years after the economic shock and only in a limited number of countries. To address this limitation, we assess the impact of the economic lockdown on household incomes by means of simulating counterfactual scenarios with a fiscal microsimulation approach (Figari *et al*., 2015).

First, we use pre‐pandemic household income surveys to construct our baseline income distribution before the pandemic. We then use timely information on earnings and employment changes during the first month of the pandemic in each country, based on COVID‐19‐related surveys or policy legislation. We use these timely data to simulate similarly sized earnings and employment shocks to workers in the pre‐pandemic household surveys. Workers affected by the shocks become unemployed, furloughed or see a reduction in their hours/earnings. Second, we use the tax‐benefit model EUROMOD to calculate household tax liabilities, benefit entitlements and net incomes in the baseline and after the simulation of shocks. Thus, we consider the direct cushioning effect of the tax‐benefit system which depends on household market incomes as well as individual and household characteristics. It is important to note that we do not consider other aspects such as the reduced likelihood to get a job for those who are looking for one and the wider consequences of macroeconomic feedbacks. The use of microsimulation models to consider how welfare systems protect people against an extreme shock is known as a “stress test” of the tax‐benefit system (Atkinson, 2009) and has become increasingly popular in analysing consequences of the Great Recession as shown for instance by Fernandez Salgado *et al*. (2014) and Jenkins *et al*. (2013).

A key feature of our analysis is that it provides a first cross‐country analysis of the initial impact of the COVID‐19 pandemic on household net incomes, taking into account the interaction between the country‐specific economic shocks and the national policy responses. In particular, we find that cross‐country variations in the budgetary and distributional consequences of the pandemic depend on (1) the asymmetric dimension of the shock in each country, (2) the different levels of protection offered by the tax‐benefit systems, (3) the diverse design of discretionary measures and (4) the differences in the household level circumstances and living arrangements of individuals at risk of income loss in each country.

The paper is structured as follows. Building on a review of the most up‐to‐date contributions to the literature, we highlight the main motivations for our approach in Section 2. There we describe the tax‐benefit model EUROMOD; simulations of the COVID‐19 earnings and employment shocks and the characteristics of those affected by the shocks in the different countries considered; and the indicators we apply to measure the resilience of the welfare systems in both relative and absolute terms. The most relevant features of the policy measures included in the analysis are described in Section 3. Empirical evidence on the size and distribution of earnings losses and compensation offered by the countries’ tax‐benefit systems is presented in Section 4. Section 5 shows the differing degrees of relative and absolute welfare resilience across countries. In Section 6 we conclude, summarize the main findings and suggest future improvements in light of ongoing developments as data are made available.

## 2 Literature Review and Empirical Approach

As Clark *et al*. (2020) have recently underlined, we observe a fast‐growing literature on the impact of lockdowns on well‐being (e.g. Layard *et al*., 2020; Brodeur *et al*., 2021), labor market participation (e.g. Adams‐Prassl *et al*., 2020), levels of unemployment or underemployment (e.g. Guven *et al*., 2020) and gender equality (e.g. Alon *et al*., 2020; Farré *et al*., 2020) in a variety of countries.

Regarding the distributional consequences of the pandemic, Clark *et al*. (2020), Menta (2021) and Belot *et al*. (2020) provide a cross‐country comparative perspective exploiting two specific surveys undertaken in different countries. The first two papers use longitudinal high‐frequency information on household disposable income in five European countries (France, Germany, Italy, Spain and Sweden) run by the University of Luxembourg starting at the end of April 2020 and the third one uses cross‐sectional data from China, Japan, South Korea, Italy, the UK and the US also for April 2020. However, the data do not allow to distinguish the impact of the labour income shocks versus the extent to which the tax‐benefit systems and emergency policies have provided household income stabilisation.

Several country‐specific studies specifically focus on the impact of welfare measures on household disposable income: Beirne *et al*. (2020) and O’Donoghue *et al*. (2020) on Ireland, Brewer and Gardiner (2020) and Brewer and Tasseva (2021) on the UK, Bruckmeier *et al*. (2020) on Germany, Figari *et al*. (2020) on Italy, Li *et al*. (2020) on Australia and Marchal *et al*. (2021) on Belgium. Nevertheless, using the results of different country‐specific analyses to compare the levels of welfare resilience to a shock is a challenging task because authors use diverse strategies to simulate the impact of the shocks on household incomes and analyse different indicators of resilience.

Almeida *et al*. (2020) present EU cross‐country comparisons for the impact of the pandemic on household incomes, using a re‐weighting approach to simulate COVID‐19 labor market shocks. They start from the macroeconomic scenarios included in the European Commission Spring 2020 forecasts and translate the changes in several aggregate variables present in the macroeconomic scenarios into changes at the individual level by reweighting individual observations. This reweighting strategy is useful but has a main drawback as it assumes that the new unemployed or furloughed have similar characteristics as those observed in the data and does not adjust to real sector‐specific unemployment changes due to lockdown nor does take into account newly introduced emergency measures, ignoring the potentially heterogeneous effect of these schemes across the income distribution.

We depart from the above‐mentioned studies by applying the so‐called stress‐testing approach to tax‐benefit systems in order to assess the impact on household incomes of the COVID‐19 shocks and government responses at the onset of the pandemic in Belgium, Italy, Spain and the UK. Our contribution is novel in that we provide timely and meaningful cross‐country comparative evidence on the distributional impact of COVID‐19 and the governments’ policy responses, adopting a fully individual micro‐level analysis comparable across countries.

### 2.1 Stress‐testing the Tax‐benefit Systems

The COVID‐19 pandemic and the containment measures created a sudden economic shock with a direct impact on the labour market participation of individuals, and hence on household incomes. To inform policy learning, it is essential to provide timely analysis to assess the success of the existing as well as emergency tax‐benefit and earnings compensation schemes in protecting household incomes. The fiscal literature refers to the first as automatic stabilizers and to the latter as discretionary policies (Paulus and Tasseva, 2020). Unfortunately, micro‐data on household incomes during the pandemic will only become available with a few years lag. To address this data limitation, we simulate labor market COVID‐19 shocks, and predict household incomes before and during the crisis, by combining a tax‐benefit model with different sources of household micro‐ and/or macro‐data.

Our method follows closely the approach to stress‐test the welfare state. Stress‐testing is commonly used to assess the vulnerability of portfolios in financial institutions and the resilience of the financial systems to extreme, but plausible shocks (Jones *et al*., 2004). Atkinson (2009) suggests extending the approach to tax‐benefit systems to assess their resilience to major economic downturns. Stress‐testing can be applied to assess the effects of either hypothetical or contemporary shocks for which no household micro‐data are yet available. Thus, we follow the latter (contemporary shocks) option and build on the work by Fernandez Salgado *et al*. (2014), who analyze the income compensation provided by the welfare state to newly unemployed at the onset of the Great Recession. Bruckmeier et al. (2020) is a recent application of the same approach implemented through a combination of data and models on firm output expectations, labor demand and individual level policies.

For the individuals affected by the simulated shocks, we analyse how much of the loss to their labor income is cushioned by the existing fiscal policies—that is, automatic stabilizers—in each country in the form of: (a) income taxes and social insurance contributions, (b) contributory benefits for those who lost their earnings, (c) other means‐tested benefits and tax credits designed to protect families on low income, and (d) other household incomes, in the form of earnings of those still in work as well as capital incomes, pensions and benefits, received by other household members. In addition, we capture the distributional effects of the new emergency policies governments implemented to prevent the sudden fall in household income.

With our stress‐testing approach we focus exclusively on the loss of earnings as one of the channels through which the COVID‐19 pandemic directly affects individual well‐being and on the direct compensation provided by the tax‐benefit system and earnings compensation schemes. We abstract from other potential adaptive changes in individuals’ and their family members’ behavior and from general equilibrium consequences in the short or long term. Thus, we assess the extent to which the welfare system helps to stabilize household incomes across countries, and whether there are specific weaknesses in the policy instruments in operation.

### 2.2 Counterfactual Scenario Derived Using EUROMOD

We make use of household micro‐data and a tax‐benefit microsimulation model to estimate baseline household incomes, that is, before COVID‐19, and counterfactual household incomes during the first month (i.e. April 2020) of the pandemic.

To derive our baseline scenario of the pre‐COVID‐19 income distribution, we use household micro‐data from the Statistics on Income and Living Conditions (SILC) for 2018 (with 2017 incomes) for Belgium, Italy and Spain and from the Family Resources Survey (FRS) for 2018/19 for the UK. Both the SILC and FRS include very rich information on individual and household characteristics and incomes and are broadly representative of the national population before the onset of the pandemic. The financial values of the income data are uprated to 2020 to account for the average growth in earnings and statutory indexation of public pensions and disability benefits between 2017/2018 and 2020. We do not make any adjustments for changes in the population composition between 2017/2018 and 2020. We then combine these data on gross (pre‐tax) market/original incomes with the European tax‐benefit model EUROMOD which calculates for each individual/household in the sample their social insurance contributions (SIC), income tax liabilities and benefit entitlements, as well as their disposable income, based on the 2020 pre‐COVID‐19 tax‐benefit rules.

To the extent it is relevant in each country, EUROMOD baseline simulations are corrected for income tax evasion (Italy) and benefits non‐take‐up (UK, Belgium) and we assume there are no changes in the tax evasion and benefit take‐up behavior as a consequence of the shock.

For more information on EUROMOD, see Sutherland and Figari (2013) and the EUROMOD Country Reports (Assal *et al*., 2020 for Belgium; Ceriani *et al*., 2020 for Italy; Navas‐Román and Villazán‐Pellejero, 2020 for Spain; and Reis and Tasseva, 2020 for the UK) for the details on the policies simulated.

To derive our counterfactual scenario, we simulate employment and earnings shocks to the workers in the SILC and FRS samples. These shocks resemble the COVID‐19 shocks that occurred in the first month of lockdown in each country. The shocks simulations are informed by the most up‐to‐date (at the time of writing) and detailed information on the labor market changes in each country, based on: external micro‐data from the Corona Study for Belgium; information on the economic sectors enforced to shut down by the national laws in Italy; the Labour Force Survey and aggregate statistics from the social security registers for Spain; the Understanding Society COVID‐19 Study for April 2020 for the UK. To simulate the shocks as accurately as possible, we apply somewhat different approaches in each country, taking into account differences in the types of shock and available data. Despite differences in the approaches, in all four countries we focus explicitly on modeling changes to (self‐) employment and earnings and household incomes, which allows us to draw consistent and meaningful cross‐country comparisons.

In more detail, we apply the following approaches to simulate the COVID‐19 labor market shocks:

In Belgium, we identify affected individuals by calculating the propensity of workers to become temporary unemployed or to have to shut down their self‐employment activities. The characteristics that define this propensity are derived from an analysis of the Corona study, a survey that tracks the experiences of households during the lockdown and its aftermath. The propensity is calibrated against administrative data on the share of employees receiving a temporary unemployment benefit or a bridging right for self‐employed in the month of April, by sector, age group and gender. More details on the method used can be found in Marchal *et al*. (2021).

In Italy, we identify workers in the economic sectors at 6‐digit ATECO level that were listed in the Decree Law imposing the shutdown of economic activities.^2^ Although SILC microdata lack information on business activities at 6‐digit level, we draw on other detailed available statistics released by Istat—namely, the operating firms archive (ASIA), the national labor force survey (RCFL) and National Accounts—in order to compute the occupation shares in each sector subject to shut down. We then randomly select the individuals, with a positive income source from either employment or self‐employment. We perform this selection by sector of employment at 2‐digit ATECO level, which we relate to data in EUROMOD in order to get the same occupation shares subject to the shutdown. Details can be found in Figari and Fiorio (2020).

In Spain, we estimate the propensity of active male and female adults to become unemployed using the 2018 Spanish Labour Force Survey (SLFS) by age, education, civil status, household type, immigrant origin, activity status, industry or sector, occupation and region. We use the estimated coefficients from the probit model to predict the probability of an unemployment outcome for each employed individual in the SILC sample and randomly assign each individual to one of the outcomes (unemployment or employment) respecting these probabilities. We then order individuals by sector and region and according to this random assignment we are able to calibrate our numbers by the real impact of the shock using social security registers for the months of March and April 2020 by sector (12 categories) and region (7 categories). Moreover, we randomly select workers entering furlough by sector of activity and region and calibrate against administrative data the share of employees or self‐employed receiving a temporary unemployment benefit in the month of April.

In the UK, we use data from the April 2020 wave of the Understanding Society COVID‐19 Study (UKHLS hereafter) which contains information on individuals’ labor market status and earnings in February 2020 (before COVID‐19) versus April 2020 (after COVID‐19). We first estimate two multinomial logit models on the UKHLS data—one on the sample of employees and another one on the sample of self‐employed, both with positive earnings in February 2020. The model for employees has four outcomes: (1) out of work, (2) furloughed, (3) still employed but with reduced hours and earnings, (4) still employed and with no drop in earnings. Similarly, the model for self‐employed has three outcomes: (1) out of work, (2) still self‐employed but with reduced hours and earnings, (3) still self‐employed and with no drop in earnings. In both models, we control for a range of individual and household‐level characteristics, including age, sex, industry, household type, baseline earnings quintile/ventile groups and number of working hours in bands by sex. We then take the estimated coefficients from the models and apply them on the sample of FRS workers with positive earnings, to predict the probability of each labor market outcome for each worker. We randomly assign each worker to one of the outcomes, accounting for these predicted probabilities. For detailed information on the approach and estimates from the multinomial logit models, see Brewer and Tasseva (2021).

Finally, using EUROMOD, we apply the tax‐benefit rules as of April 2020 on the data with modified workers’ earnings to compute SIC, taxes, benefits, earnings compensation schemes and household disposable incomes during the first month of the COVID‐19 crisis. By comparing the baseline and counterfactual, we estimate the impact on household incomes of the crisis and governments’ fiscal policies. We discuss the tax‐benefit and earnings compensation schemes in more detail in Section 3.

### 2.3 The Characteristics of those Affected by Earnings Loss

The analysis focuses on employed and self‐employed individuals who lost (all or part of) their earnings in the immediate aftermath of the COVID‐19 outbreak. The proportion of workers simulated with earnings losses ranges from 20 percent in Spain to around 30 percent in Belgium and Italy and 37 percent in the UK (Table 1).

**TABLE 1:** Characteristics of those Affected by Earnings Losses

	Belgium	Italy	Spain	UK
*Workers affected by earnings losses %*	30.28	28.88	19.85	37.46
*Presence of children %*	41.5	39.05	38.75	41.89
*Number of earners in the family %*				
1	31.75	40.73	26.77	24.22
2	56.11	44.15	51.65	54.09
3+	12.14	15.11	21.59	21.69
*Household income quintile %*				
Bottom	8.50	13.64	15.01	11.63
2nd	17.76	15.39	19.80	16.83
3rd	22.65	20.93	21.29	21.98
4th	25.44	24.74	23.11	25.82
Top	25.66	25.31	20.79	23.75

Notes:

Summary statistics for those affected by earnings losses as identified in EUROMOD data. Quintile groups based on equivalized household disposable income in the baseline.

Around 40 percent of those affected by an earnings shock live in a family with children, pointing to the need to have welfare systems that protect not only workers but their dependent family members as well. Moreover, the share of those being the only earner in the family is relatively high ranging from around 25 percent in Spain and the UK to 32 percent in Belgium and 41 percent in Italy: for their families the temporary shutdown of their activities implies the loss of the main income source.

The distribution of those affected by an earnings shock by household net income quintile groups (assessed before the earnings loss) shows an increasing pattern despite important differences across countries. There are relatively less individuals affected in the first quintile in Belgium, the UK and Italy than in Spain where the distribution across quintiles is more uniform.

### 2.4 Income Stabilization Indicators

Following Fernández Salgado *et al*. (2014), we focus on three indicators measuring the relative and absolute resilience provided by the tax‐benefit policies and earnings compensation schemes during the pandemic. These indicators are the Net Replacement Rate, the Compensation Rate and changes to the Poverty Rate.

The Net Replacement Rate is a measure of relative resilience and captures the level of income stabilisation with respect to the baseline income (Immervoll and O’Donoghue, 2004). It is computed on the sample of workers affected by the shock and equals:

$$\text{Net Replacement Rate}=\frac{Y_{\text{post}}}{Y_{\text{pre}}}$$

where *Y*_pre_ and *Y*_post_ are equivalized household disposable income in the baseline and after the shock, respectively. Household disposable income is made up of the sum of gross (pre‐tax) original income (i.e. earnings from (self‐)employment, private pensions, private transfers, income from rent and investment income) and public benefits minus taxes (i.e. income taxes plus SIC). To account for household composition and economies of scale within the household, household incomes are equivalized using the modified OECD equivalence scale (a value of 1 for the head, 0.5 for any other adult aged 14+ and 0.3 for each child aged <14).

To measure the level of income protection provided by the different policies, we break down the Net Replacement Rate by income source:

$$\text{Net Replacement Rate}=\frac{O_{\text{post}}+B_{\text{post}}‐T_{\text{post}}}{Y_{\text{pre}}}$$

where *O*_post_, *B*_post_ and *T*_post_ are equivalized household original income, public benefits and taxes, respectively, after the shock. We also break down further *B*_post_ into the different benefit types to show explicitly the contribution of (1) the earnings compensation schemes, (2) unemployment benefits and (3) means‐tested and other benefits (e.g. housing and social assistance benefits).

The Net Compensation Rate is another indicator of relative resilience which captures the level of protection offered by fiscal policies. It is also computed on the sample of workers affected by the COVID‐19 shocks and measures the proportion of net earnings lost due to the crisis, compensated by public benefits net of taxes, as follows:

$$\text{Net Compensation Rate =}\;\frac{\left(B_{\text{post}}‐B_{\text{pre}}\right)\;‐\left(T_{\text{post}}^{\text{*}}‐T_{\text{pre}}^{\text{*}}\right)}{\;\left(E_{\text{post}}^{\text{*}}‐E_{\text{pre}}^{\text{*}}\right)\;},$$

where *B*_post_ and *B*_pre_ are equivalized household public benefits, $T_{\text{post}}^{\text{*}}$ and $T_{\text{pre}}^{\text{*}}$ are taxes liable on the worker’s own earnings and $E_{\text{post}}^{\text{*}}$ and $E_{\text{pre}}^{\text{*}}$ are the worker’s earnings net of taxes, respectively, after and before the shock. Thus, the denominator captures the loss in *net* earnings due to the shock, while the numerator shows how much of this loss is absorbed by more generous benefit entitlements and/or lower taxes. The Net Compensation Rate allows us to isolate the net government support, abstracting from the income insurance provided by the gross original income of other household members. As with the Net Replacement Rate, we break down further the Net Compensation Rate to show the contribution of benefits by type.

Our indicator of absolute resilience is the change in the Poverty Rate due to the shock measured against a *fixed* poverty threshold, that is, 60 percent of the median baseline equivalized household disposable income. We look at poverty changes among different subgroups (workers affected by the shock and children) versus the total population. Our approach of using a poverty threshold fixed to the baseline income distribution follows the suggested practice to measure poverty during an economic downturn with a poverty line fixed in real terms (Jenkins *et al*., 2013). It allows to capture the drop in living standards that individuals face, by comparing their current circumstances with their situation before the income shock (Matsaganis and Leventi, 2011). A normative judgment about the appropriate level of income protection provided by the welfare state is beyond the scope of this paper (Boadway and Keen, 2000). Nevertheless, given the overarching policy objective of limiting the number of individuals at risk of poverty, it is implicit that household incomes should not fall below the poverty threshold as a result of the crisis.

Finally, when interpreting the results, it should be noted that our main indicators—the Net Replacement Rate, Compensation Rate and changes to the Poverty Rate among workers affected by the shock—are estimated for the group of workers affected by the COVID‐19 shocks only and thus, results are not affected per se by the number of individuals affected by the shocks. In comparison, the proportion of affected workers matters for total population estimates of the budgetary costs and changes to income poverty and inequality.

## 3 Income Protection Policies During COVID‐19

The existence in all European countries of a developed welfare state, that is intended, among other things, to protect people and their families against economic shocks, is one of the main differences between the crisis faced today and that of the 1930s. However, the sudden and unexpected shock due to the COVID‐19 pandemic forced European governments to adapt existing measures and to define new discretionary and bold measures to support those who are bearing a disproportionate share of the economic burden (OECD, 2020a).

Table 2 provides a summary of the most important measures implemented in April 2020 in our selection of countries. All four countries took similar provisions to safeguard incomes of employees, though the design, size and scope is somewhat different. In what follows we refer to “Earnings Compensation Schemes” to identify the different instruments (i.e. furlough schemes and subsidies to self‐employed) in place in each country to protect employment and self‐employment incomes (Konle‐Seild, 2020).

**TABLE 2:** Governments Responses to the COVID‐19 Pandemic (Simulated Policies Only)

Belgium	Italy	Spain	United Kingdom
*Earnings compensation schemes—employees*			
Temporary unemployment benefit [*Tijdelijke werkloosheid—COVID‐19*]	Temporary unemployment benefit *[CIG = Cassa Integrazione Guadagni]*	Temporary unemployment benefit *[ERTE = Expediente de Regulación Temporal de Empleo]*	Coronavirus Job Retention Scheme
Existing scheme extended to all employees	Existing scheme extended to most employees	Existing scheme	New scheme
Up to 70% with min and max (€2,084)	Up to 80% with max (€1,130)	Up to 70% with min and max (€1,411)	Up to 80% with max (£2,500)
*Earnings compensation schemes—self‐employed*			
Bridging right [*Tijdelijke crisismaatregel overbruggingsrecht*]	*Lump sum transfer [Bonus €600]*	Temporary unemployment benefit *[Prestación extraordinaria por cese de actividad COVID‐19]*.	n/a
Existing scheme extended	New scheme	New scheme	
Lump‐sum benefit (€1,262/ €1,614)	Lump sum benefit (€600)	Up to 70% with min and max (€1,411)	
*Unemployment benefits*			
Contributory unemployment benefit for employees *[Werkloosheidsverzekering]*	Ban for firms to lay off employees	Contributory unemployment benefit for employees *[Prestación por desempleo]*	Contributory unemployment benefit for employees [contribution‐based Jobseeker’s Allowance]
		Contributory unemployment benefit for self‐employed *[Prestación económica cese de actividad de trabajadores autónomos]*	
*Other schemes*			
Social assistance benefit *[Leefloon—Recht op maatschappelijke Integratie]*	Lump sum transfer (€100) to employees working at their firms’ premises		Universal Credit (main allowances increased by £20 per week, rent component increased); Working Tax Credit (main allowances increased by £20 per week), Housing Benefit (increased, earnings disregard increased by £20 per week), Council Tax Reduction (earnings disregard increased by £20 per week)

Notes:

Authors’ elaborations based on national legislation. See the EUROMOD Country Reports for details.

Starting with the provisions for employees, the most important measure in Belgium was the extension of access to the so‐called system of temporary unemployment to all employees (“*Tijdelijke werkloosheid COVID‐19*,” a furlough‐type measure); previously, the system was only accessible for economic conditions or under “force majeure,” but under COVID‐19 a much broader definition was applied and the application procedure was considerably simplified, causing most employees from impacted employers to be eligible. Benefit generosity was increased on the one hand by raising the replacement rate from 65 percent to 70 percent of the previous monthly wage (with lower and upper bounds) and on the other hand by providing a daily supplement of €5.63. The daily benefit ranges from a minimum of €55.59 up to a maximum €74.71, and is paid according to a 6‐day work week. In April a withholding tax of 26.75 percent was applied at source. Alternatively, unemployed workers can get the existing contributory unemployment benefit.

To compensate the earnings loss suffered by the employees in Italy, the government extended with the Decree Law 18/2020 (“Cura Italia”) the existing furlough scheme (i.e. *Cassa Integrazione Guadagni,* CIG) relaxing the eligibility conditions and allowing most of employees to be entitled to the scheme. Only domestic workers and consultants (i.e. *parasubordinati*) are not eligible. The wage compensation scheme provides a replacement of 80 percent of earnings subject to a maximum cap: if monthly earnings are below €2,160, *CIG* cannot exceed €940, while if earnings are above the threshold the *CIG* is capped at €1,130. This implies that in practice the replacement rate can be substantially below 80 percent for most workers. Transfer payments are subject to income taxes. The same Decree Law imposes that firms cannot fire employees after February 23, 2020: this implies that existing Unemployment Insurance Schemes do not apply to the generality of workers but only to those with temporary contracts that reach the end during the COVID‐19 pandemic.

In Spain, the Contributory Unemployment benefit (*Prestación por desempleo*) and the Temporary unemployment subject to administrative approval (*Expediente de Regulación Temporal de Empleo*, a furlough‐type measure) also provide protection through a replacement rate of 70 percent with lower and upper bounds. Similarly to the Belgian case, this temporary unemployment system was already in place but was only accessible for economic conditions or under “force majeure,” but under COVID‐19 a much broader definition was applied and the application procedure was considerably simplified, causing most employees from impacted employers to be eligible. The benefit can range between €502 and €1,411 per month.

To support business and workers in the UK, the government introduced a new Coronavirus Job Retention Scheme to subsidize earnings of furloughed employees. The scheme allows employers to reduce employees’ working hours to zero, without laying employees off and thus, reducing the costs of searching and re‐hiring workers later on. In April 2020, this scheme pays 80 percent of gross earnings up to a maximum of £2,500 per month. In the case of a job loss, employees who have previously paid SIC are entitled to the contributory unemployment benefit Jobseeker’s Allowance (JSA).

Belgium, Italy and Spain also set up provisions for the self‐employed (the UK Self‐employment Income Support Scheme was introduced from May 12 onwards and is hence not considered in this analysis^3^). In Belgium this was done through an extension of access to the so‐called bridging right (“*Overbruggingsrecht”*). It entails a lump‐sum transfer of €1,291.69 and €1,614.10 per month for self‐employed without and with dependent family members, respectively. Amounts are halved for those self‐employed whose activity is a secondary one and whose yearly income ranges between €6,996.89 and €13,993.77. To compensate the earnings loss incurred by the self‐employed in Italy, the government defined a new lump‐sum transfer of €600 to be paid for the month of March to *all* self‐employed, irrespective of whether they incurred a loss or not. The self‐employed in specific professional bodies (e.g. lawyers, accountants, notaries, etc.) are eligible for the lump‐sum transfer only if their 2019 income was below €35,000. The transfer is not subject to income tax and does not enter in any means‐test of other benefits. In Spain a contributory unemployment benefit for the self‐employed was already in place since November 2010. The Spanish government however, set up additional provisions for the self‐employed through a temporary unemployment benefit linked to the COVID‐19 crisis. This benefit has the aim to protect earnings of those self‐employed who did not have enough contributions (12 months prior to shock) to be eligible for the contributory unemployment benefit. Both benefits provide protection through a replacement rate of 70 percent with lower and upper bounds.

In Italy, employees bound to continue work on company premises and those who cannot typically work from home are entitled to a lump‐sum transfer of €100 to be paid for the month of March. Estimates show that 50 percent of employees working in the economic sectors that are not subject to the shutdown still work on company premises (Fondazione Studi Consulenti del Lavoro, 2020). The transfer is not subject to income tax and does not enter in any means‐test of other benefits.

With the exception of income tax liabilities, which are expected to go down due to the lower level of earnings, the rest of traditional automatic stabilizers embedded in the tax‐benefit systems operate in a different way across countries.

SIC paid by employees and self‐employed fall because of the losses to earnings and because either they are calculated based on the amount of benefits replacing lost earnings by less than 100 percent as in the UK or because they are credited by the government (and as such not accounted for in this analysis) as in Belgium and Italy. The exception is Spain where only employer SIC are credited by the government and workers are still paying contributions on the same base as with previous earnings.

The existing income‐tested benefits in Italy (i.e. the bonus IRPEF, Family allowances and the Citizenship income) and Spain (i.e. the Regional minimum income schemes—*Rentas Mínimas*) are based on the income and means‐test of the previous fiscal year—or at least previous months—and thus, do not react immediately to the loss of earnings households experienced in April 2020. In contrast, in the UK, entitlements to the income‐tested benefits are calculated based on “current” incomes and circumstances, allowing them to compensat e families immediately for the income losses. In addition to accessing the unemployment benefit JSA, low‐income families and/or unemployed individuals can receive support from the main means‐tested benefit Universal Credit (UC) which consists of a standard allowance and additional allowances depending on the person’s and their family’s circumstances. Before COVID‐19, UC and JSA paid the same amount to single individuals aged 25+ of £323 per month. In response to the pandemic, the UK government increased UC standard allowance by £20 per week which was a significant increase in relative terms of 28 percent for singles aged 25+ and 17 percent for couples. Access to UC for self‐employed was also relaxed. The UC allowance which supports families paying their rent and Housing Benefit (HB) were made more generous by increasing the Local Housing Allowance Rates used to calculate benefit entitlements. Income support is also provided to low‐income families by other means‐tested benefits such as Working Tax Credit (WTC) and Council Tax Reduction (CTR). The basic allowance of WTC was increased by £20 per week in line with UC. Finally, the earnings disregard for HB and CTR was increased making benefits more generous. Noteworthy, the increased benefit generosity in the UK will have an impact not only on the incomes of families affected by the COVID‐19 shocks but also on those already claiming benefits prior to the pandemic. In Belgium, no changes occurred to the means‐tested social assistance benefit scheme in the immediate aftermath of the lockdown. Eligibility to the benefit depends on current need (and assets). In principle, social assistance can act as a top‐up to those who saw their income decrease under the social assistance threshold.

In addition to the policies listed in Table 2, other policy changes have been introduced, but these are not included in the analysis mainly due to data unavailability. Examples of these are: in Belgium the degressivity of unemployment benefits was suspended. In Italy, the government allowed employees in the private sector with children up to 12 years old to take parental leave for 15 days at 50 percent of the earnings’ level or, alternatively, to have a babysitting bonus of €600 (incremented to €1,000 for those working in the health system). In Spain, there was rent payment help, as well as an extra subsidy for domestic workers and temporary workers. In addition, we do not consider the suspension of mortgage payments on the main residence in Belgium, Italy and Spain because these policies involve only a change in the timing of payment with potential effects on lower than usual interest rates for these payments.

## 4 Budgetary Effects and Distributional Changes

We first show the simulated fiscal cost of the main income protection schemes acting in each country at the onset of the pandemic. We then assess the impact of COVID‐19 and the policy responses on household gross (pre‐tax) original and disposable income and the share of gainers and losers, on average for the whole population and by income quintile groups of pre‐COVID‐19 incomes. Finally, we analyze the impact of the crisis on income inequality.

Table 3 reports the simulated costs and the number of entitled individuals for each policy measure compared with the available figures from administrative statistics. Several caveats need to be considered as the comparison between simulated and administrative figures is particularly challenging due to the lack of availability of administrative figures at the same detailed level and for the same time period as considered in the paper, that is, April 2020. Nevertheless, this comparison of the available figures shows the generally high level of external validity of our simulations, in particular related to the main simulated instruments.

**TABLE 3:** External Validity of Simulations

	Simulations			Administrative Data	
	Cost		Entitled	Cost	Entitled
Country and Policy	Billions	% of Annual GDP	Thousands	Billions	Thousands
*Belgium*					
Earnings compensation scheme—employees	1.3	0.26	1,117	1.3	1,170
Earnings compensation scheme—self‐employed	0.4	0.08	278	0.57	405
*Italy*					
Earnings compensation scheme—employees	4.8	0.27	5,566	n.a.	5,500
Earnings compensation scheme—self‐employed	1.9	0.11	3,230	2.4	3,955
Lump sum transfer (€100)	0.6	0.03	5,968	n.a	n.a.
*Spain*					
Earnings compensation scheme—employees	2.4	0.20	2,788	2.5	2,381
Earnings compensation scheme—self‐employed	0.7	0.06	1,036	1.233	1,138
Unemployment benefit—employees	0.5	0.04	424.6	n.a.	n.a.
Unemployment benefit—self‐employed	0.04	0.00	57.3	n.a.	n.a.
*UK*					
Earnings compensation scheme—employees	10.4	0.46	7,305	n.a.	8,787
Unemployment benefit	0.2	0.01	0.6	n.a	n.a.
Universal Credit	0.9	0.04	5,380	n.a.	5,260

Notes:

Costs and entitlements refer to 1‐month payments (April 2020). Amounts are expressed in €/EUR in Belgium, Italy and Spain; in £/GBP in the UK. GDP for 2019 based on Eurostat data (online data code: TEC00001). “Earnings compensation scheme—” includes Temporary unemployment in Belgium, CIG and lump sum benefit in Italy, ERTE in Spain, Coronavirus Job Retention Scheme in the UK. “Earnings compensation scheme—self‐employed” includes Bridging right in Belgium, Lump sum transfer in Italy, Temporary unemployment for self‐employed in Spain. In the UK, the simulated number of entitled to the “Earnings compensation scheme—employees” refers to the number of furloughed employees, while the administrative data refers to the number of employments.

Overall the resources dedicated to compensate income losses of individuals and families for the first month of the pandemic crisis ranged from 0.30 percent of annual GDP in Spain to 0.51 percent in the UK, showing large disparities in the resources allocated in response to the crisis, but in line with differences in the share of workers affected by the labor market shocks as shown in Table 1.

Across countries the measures that absorbed most resources are the earnings compensation schemes for employees, followed by the new instruments introduced to sustain self‐employed incomes. Substantial resources were also devoted to the increased generosity of the existing means‐tested benefits in the UK and to the payment of contributory unemployment benefits in Spain.

Overall, the 1‐month shutdown at the beginning of the COVID‐19 crisis implied a loss of original income of around 0.51 percent of annual GDP (€6 billion) in Spain, 0.64 percent (€3 billion) in Belgium, 0.89 percent (€16 billion) in Italy and 0.96 percent (£22 billion) in the UK. With such a loss of original income, governments lost substantial amounts of income tax revenue and SIC (including both employer and employee contributions) which act as automatic stabilizers, reducing their financial burden on the individuals who experienced an income loss. The main exception is represented by Spain where workers, while receiving the benefits, continued paying SIC calculated on the previous “contribution base” as defined while at work. Despite additional resources transferred as state benefits ranging from 0.29 percent of annual GDP in Spain to 0.52 percent in the UK, the loss of disposable income for families was between 4 percent and 5 percent of the disposable income before the shock in Belgium, Italy and Spain and around 8 percent in the UK (Table 4).

**TABLE 4:** Income Changes Due to COVID‐19 and Governments’ Policy Responses

Income Source	Billions	% of Annual GDP	% Change
*Belgium*			
Original income	−3.0	−0.64	−18.39
SIC: employer	−0.5	−0.12	−15.36
SIC: employee and self‐employed	−0.4	−0.09	−19.09
Income tax	−0.4	−0.08	−8.22
State benefits	1.7	0.35	25.76
Disposable income	−0.6	−0.12	−3.67
*Italy*			
Original income	−15.9	−0.89	−25.97
SIC: employer	−3.4	−0.19	−25.96
SIC: employee and self‐employed	−1.7	−0.09	−25.87
Income tax	−2.8	−0.15	−16.19
State benefits	8.0	0.45	27.84
Disposable income	−3.4	−0.19	−5.15
*Spain*			
Original income	−6.3	−0.51	−15.66
SIC: employer	−1.5	−0.12	−15.60
SIC: employee and self‐employed	−0.1	−0.01	−3.54
Income tax	−0.8	−0.06	−10.90
State benefits	3.6	0.29	25.41
Disposable income	−1.8	−0.15	−4.11
*UK*			
Original income	−21.8	−0.96	−23.53
SIC: employer	−0.9	−0.04	−13.34
SIC: employee and self‐employed	−1.1	−0.05	−13.97
Income tax	−2.5	−0.11	−14.21
State benefits	11.7	0.52	68.3
Disposable income	−6.5	−0.29	−7.65

Notes:

Estimates for income changes are based on non‐equivalized incomes and refer to 1‐month (April 2020) shutdown. SIC = Social Insurance Contributions. Amounts are expressed in €/EUR in Belgium, Italy and Spain; in £/GBP in the UK. GDP for 2019 based on Eurostat data (online data code: TEC00001).

**Figure 1:**
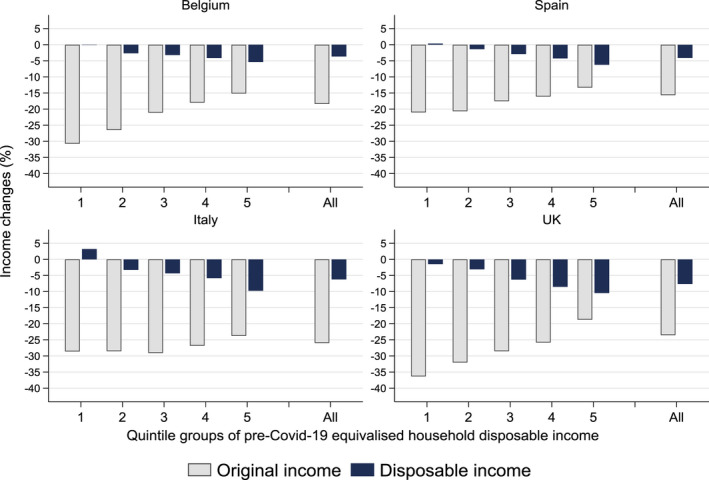
Income Changes Due to COVID‐19 and Governments’ Policy Responses, by Household Income Quintile Groups *Notes*: Changes (in %) in equivalized household original and disposable income due to COVID‐19 and governments’ policy responses. Income quintile groups are based on the pre‐COVID‐19 distribution of equivalized household disposable income. *Source*: Own calculations with EUROMOD I3.0+.

Next, Figure 1 shows the percentage change in average original and disposable income due to the crisis with respect to the baseline (pre‐COVID‐19), for the whole population as well as by quintile groups of pre‐COVID‐19 household incomes. A negative (positive) change means a loss (gain) to income. On average for the whole population, in all four countries both original and disposable income fell substantially, with the loss to original income being several times larger than the loss to disposable income. The drop to original income ranged from 16 percent in Spain to 26 percent in Italy while the drop to disposable income was from around 4 percent in Belgium and Spain to 6 percent in Italy and 8 percent in the UK.

Along quintiles groups, we see the unequal distribution of income losses. Consistently across countries, original income losses were more pronounced at the bottom of the distribution. This is in part due to the fact that one‐earner families are more concentrated at the bottom of the distribution and the pandemic caused the loss of their main source of original income. Along the income distribution, families are characterised by more earners and other income sources (e.g. property and capital income) which acted as self‐insurance. On average, those in the first (poorest) quintile group lost 36 percent of their original income in the UK, around 30 percent in Belgium and Italy and 21 percent in Spain. In comparison, those in the top (richest) quintile group lost 24 percent in Italy, 19 percent in the UK, 15 percent in Belgium and 13 percent in Spain.

In contrast to original income, changes in disposable income had the opposite pattern, with the largest losses at the top of the income distribution in all four countries, of around 10 percent in Italy and the UK and 5 percent in Belgium and Spain. In the bottom quintile, we find a small loss to disposable income of around 1 percent in the UK, no change in Belgium and Spain and a small income gain of 3 percent in Italy. Overall and across the distribution, disposable income fell by less than original income because of the protection of tax‐benefit policies which offset the large falls in earnings. We explore this in more detail in Section 5.

**Figure 2:**
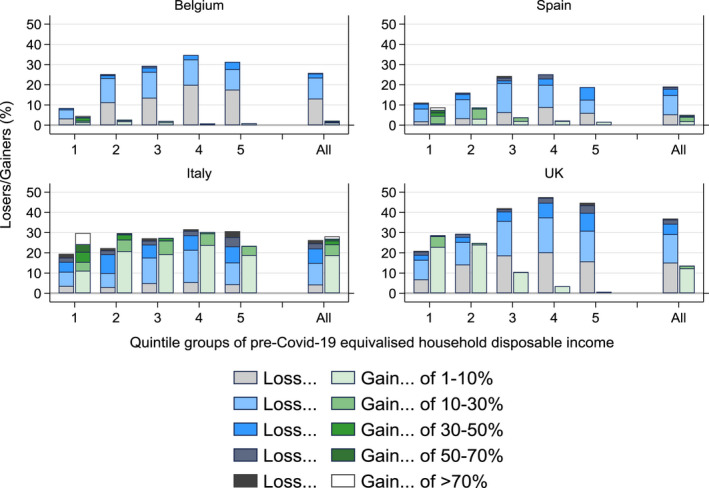
Income Gainers and Losers Due to COVID‐19 and Governments’ Policy Responses, by Household Income Quintile Groups *Notes*: Loss/gain measured as the % change in equivalized household disposable income due to COVID‐19 and governments’ policy responses. Income quintile groups are based on the pre‐COVID‐19 distribution of equivalized household disposable income. *Source*: Own calculations with EUROMOD I3.0+.

Figure 2 reports the share of individuals who experienced a loss or gain in household disposable income in the first month of the pandemic, for the whole population as well as by quintile of pre‐COVID‐19 household disposable income. We look at individuals with losses or gains of 1 percent–10 percent, 10 percent–30 percent, 30 percent–50 percent, 50 percent–70 percent and more than 70 percent of baseline (pre‐COVID‐19) disposable income. With the exception of Italy, the share of losers due to the crisis was larger than the share of gainers. In Italy, there were slightly more gainers than losers, but this was primarily due to a large group of individuals with a small gain of disposable income of between 1 percent and 10 percent. Excluding this group, there were more losers than gainers with an absolute income change of more than 10 percent.

Looking across the income distribution, in all countries there was a larger share of losers in the top three quintile groups than in the bottom two quintiles. The share of losers with losses of more than 30 percent was also higher in the middle/top than at the bottom of the distribution in Belgium and the UK. In Italy and the UK, there was also a substantial number of gainers. In Italy this was mainly due to the bonus of €100 distributed to all employees working at the firms’ premises. In the UK, the presence of gainers (with gains of 1 percent–10 percent in the bottom three quintiles and of 10 percent–30 percent in the first quintile) was due to the increased generosity of means‐tested benefits.

Despite these relevant and pronounced income changes at the top of the distribution, that also hid re‐rankings as individuals moved along the distribution due to the loss in their earnings, the Gini index based on disposable income was not statistically significantly different before and after the crisis in all countries but in Italy where we observe a non‐negligible increase in inequality (Table 5).

**TABLE 5:** Income Inequality: Gini Index

Country	Pre‐COVID‐19	Post‐COVID‐19
Belgium	0.225	0.223
	(0.004)	(0.004)
Italy	0.326	0.332
	(0.003)	(0.003)
Spain	0.322	0.320
	(0.003)	(0.003)
UK	0.309	0.306
	(0.003)	(0.003)

Notes:

Income inequality based on equivalized household disposable income. Post‐COVID‐19 refers to income inequality in April 2020. Bootstrapped standard errors after 200 replications are shown in parenthesis.

We also estimate the Atkinson index of inequality (with aversion parameter of 1) pre‐ and post‐COVID‐19 and decompose it into within and between group inequality (Table 6). Within group inequality here is the weighted sum of inequality within ventile groups of household disposable income. Between group inequality captures inequality between ventile groups if each person had the mean income in the ventile they belonged to. Consistent with the results on Gini, we estimate that only in Italy the Atkinson index increased slightly. Decomposing the index by ventile groups, not surprisingly we find that most of inequality across countries is explained by between group rather than within group inequality. In all four countries, within group inequality increased while between group inequality went down (in Italy the increase in the former was only partly offset by the decrease in the latter, explaining the overall rise to inequality). The increase in within group inequality was due to the asymmetric nature of the crisis: people’s incomes within ventiles became more heterogeneous depending on whether they were hit by the shock, how much income protection they received from the state, how many earners and what other incomes there were in the household. In contrast, the reductions to household disposable income which were largest for previously higher‐income families compressed the income distribution and reduced between group inequality.

**TABLE 6:** Income Inequality: Atkinson Index

Country	Pre‐COVID‐19			Post‐COVID‐19		
	A(1)	Within	Between	A(1)	Within	Between
Belgium	0.089	0.008	0.082	0.087	0.013	0.075
	(0.004)	(0.002)	(0.003)	(0.005)	(0.002)	(0.003)
Italy	0.176	0.011	0.167	0.186	0.05	0.143
	(0.003)	(0.001)	(0.003)	(0.003)	(0.002)	(0.003)
Spain	0.175	0.006	0.169	0.171	0.019	0.155
	(0.004)	(0.001)	(0.004)	(0.004)	(0.001)	(0.004)
UK	0.152	0.01	0.143	0.15	0.029	0.125
	(0.003)	(0.001)	(0.002)	(0.003)	(0.002)	(0.002)

Notes:

Groups based on ventile groups of equivalized disposable income. A(1) refers to Atkison index with parameter alfa of inequality aversion set to 1. Bootstrapped standard errors after 200 replications are shown in parenthesis.

Empirical evidence reported by Clark *et al*. (2020) and based on panel data from the COME‐HERE survey on five European countries shows that the pattern of inequality in Europe during the pandemic can be divided in two periods: since the beginning of the crisis up to May 2020 relative inequality slightly increased—on average across countries and in particular in Italy consistently with our results—before dropping back to pre‐COVID levels in September 2020.

## 5 Relative and Absolute Resilience

This Section assesses welfare resilience at the onset of the pandemic. To measure relative resilience, we estimate the Net Replacement Rates and Net Compensation Rates which capture the contribution of the tax‐benefit systems and household composition to income protection. To assess absolute resilience, we look at changes in the poverty rates. For more details on the measures, see Section 2.4.

### 5.1 Net Replacement Rate

**Figure 3:**
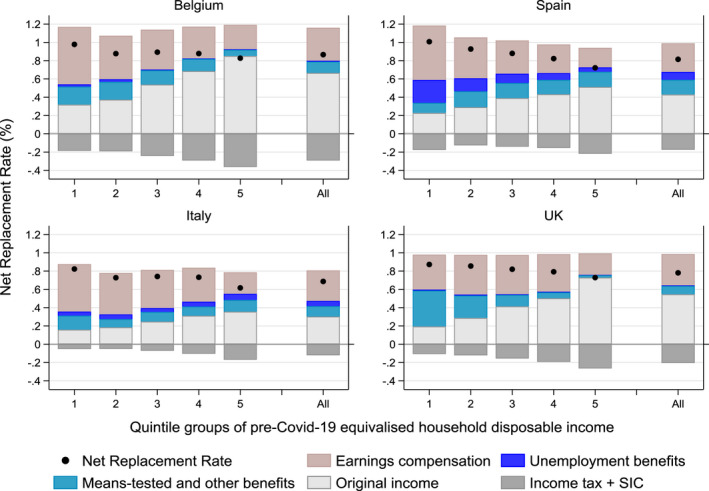
Decomposition (by Income Sources) of Net Replacement Rate for those Affected by COVID‐19, by Household Income Quintile Groups *Notes*: The Net Replacement Rate is the ratio between household disposable income after and before the COVID‐19 shocks, estimated on the sample of families affected by the shocks. All population income quintiles based on the pre‐COVID‐19 distribution of equivalized household disposable income. *Source*: Own calculations with EUROMOD I3.0+.

The average Net Replacement Rate is illustrative of the relative welfare resilience which differed across countries due to differences in tax‐benefit systems, characteristics of the individuals affected by the shutdown and household composition. Estimated on the sample of workers affected by the COVID‐19 shocks, Figure 3 shows the Net Replacement Rate (depicted as a black circle) for the whole sample as well as by quintile groups of pre‐crisis disposable income. The Net Replacement Rate is also broken down by income component (shown in bars), to show the separate contribution of: the earnings compensation schemes; unemployment benefits; means‐tested and other benefits; original income; and income tax + SIC (with the latter two reducing the Net Replacement Rates and hence shown to be negative).

Looking at the overall Net Replacement Rate, Figure 3 shows that household disposable income on average is simulated to have fallen to 87 percent of its pre‐shock level in Belgium, 82 percent in Spain and 78 percent in the UK. In Italy the Net Replacement Rate was the lowest of 69 percent, on average. Breaking down the Net Replacement Rate by income source highlights the large contribution and protective role of original income (i.e. earnings of other household members as well as other types of original income other than earnings) and the earnings compensation schemes in all countries. Post‐crisis original income (light grey bars) accounted for 30 percent of pre‐crisis disposable income in Italy, 43 percent in Spain, 55 percent in the UK, and substantial 67 percent in Belgium; while the earnings compensation schemes (light rose bars) amounted to 31 percent–36 percent across countries. Unemployment benefits (in darker blue) in Spain and Italy and means‐tested and other benefits (in lighter blue) in all four countries also contributed to protecting household incomes against the shocks, overall replacing 10 percent to 25 percent of pre‐crisis incomes. Income tax + SIC (in dark grey), as by design, reduced after‐tax incomes and accounted for −12 percent of pre‐crisis incomes in Italy, −18 percent in Spain, −21 percent in the UK and −29 percent in Belgium.

Along the income distribution, Net Replacement Rates were higher at the bottom than in the middle and top of the distribution. In Belgium and Spain, in the first quintile group the Net Replacement Rate was about 1, meaning that on average the poorest households did not see a change to their disposable income.

Across quintile groups, earnings of other household members (and income sources not affected by the economic shutdown as capital and property income) were progressively more important as household income increases: the Net Replacement Rates are likely to have been pushed up by the presence of these incomes at the top of the income distribution, but this was partly compensated by progressive income tax + SIC.

Earnings compensation schemes introduced in the different countries accounted for a substantial share of post‐shock household income across all quintiles, with a larger contribution at the bottom than the rest of the distribution in Belgium, Spain and Italy and somewhat equal contribution across the first four quintiles in the UK. In all four countries, due to the cap on earnings replacement, earnings compensation schemes protected incomes in the richest quintile the least.

Income from means‐tested benefits and other transfers (i.e. mainly pensions and disability benefits) played a smaller but important role at the bottom of the distribution mostly in Belgium and the UK, due to relative generous social assistance benefits and Universal Credit, respectively. In Spain, and to a lesser extent in Italy, an important share of family income was protected by unemployment benefits, whose impact depended on both the generosity of the schemes and number of unemployed people entitled to receive them, already higher in Italy and Spain than in other countries before the crisis.

The general lesson of our analysis is that it is necessary to consider the social protection system as a whole and how it interacts with household composition and incomes received by other household members which act as self‐insurance mechanisms. Focussing exclusively on the new emergency measures does not provide a comprehensive picture of what happened to household incomes.

### 5.2 Net Compensation Rate

**Figure 4:**
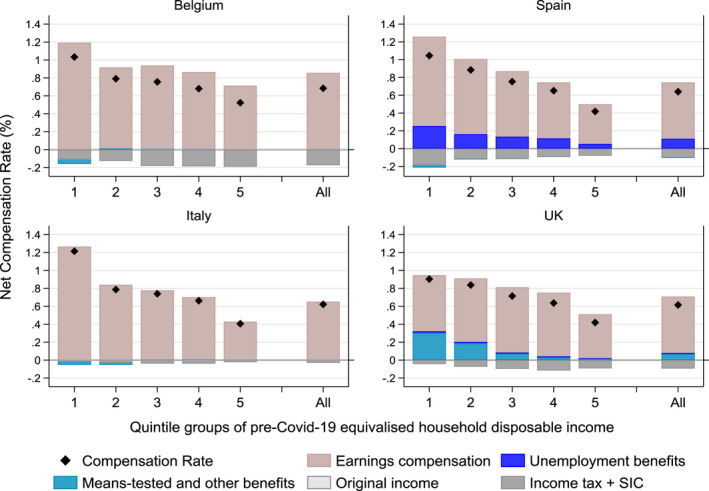
Decomposition (by Income Sources) of Net Compensation Rate for those Affected by COVID‐19, by Household Income Quintile Groups *Notes*: The Net Compensation Rate measures the proportion of net earnings, lost due to the COVID‐19 crisis, compensated by state benefits net of income tax + SIC, estimated on the sample of families affected by the COVID‐19 shocks. All population income quintile groups are based on the pre‐COVID‐19 distribution of equivalized household disposable income. *Source*: Own calculations with EUROMOD version I3.0+.

To focus on the income protection offered by public support, we now look at the Net Compensation Rate indicator. It measures the proportion of net earnings lost due to the crisis, compensated by state benefits net of income tax + SIC. As with the Net Replacement Rate, it is estimated on the sample of workers affected by the COVID‐19 shocks. Similar to Figures 3 and 4 shows the Net Compensation Rate for those affected by the shock as well as by income quintile groups (depicted by the black diamond). The Net Compensation Rate is then broken down by income source (in bars): earnings compensation schemes; unemployment benefits; means‐tested and other benefits; original income; and income tax + SIC.

Figure 4 shows that the average net public contribution to disposable income as a proportion of the net earnings lost because of the shock was the highest in Belgium with 68 percent, 64 percent in Spain, 62 percent in Italy and 61 percent the UK, with a decreasing pattern along the income distribution.

Most public support was channeled through the earnings compensation schemes (light rose bars) only slightly reduced by the income tax + SIC (grey bars) payable on some of these benefits. These schemes made up the largest share of public support at the bottom of the distribution but also provided a relatively large compensation for those in the upper part of the distribution. Families in the top quintile groups benefited relatively less than other quintiles as the amount of these schemes is capped at a maximum level in all countries, although the cap is much higher in the UK than in the other countries. In Italy and Spain other schemes are characterized either by a lump‐sum transfer (bonus €600 in Italy) or a minimum level (€502 for the temporary unemployment benefit) which increased their progressivity. Their support was less evident in the UK for the individuals at the bottom of the distribution who, however, received support from Universal Credit and other existing means‐tested benefits. In Spain, around 20 percent of earnings lost was replaced by unemployment benefits, which were relatively more important at the bottom than in the rest of the distribution.

### 5.3 Poverty Rates

The extent to which the tax‐benefit instruments allow those affected by an earnings shock to avoid falling below a given level of income depends on the generosity of the system, whether workers are entitled to receiving earnings compensation schemes, the income position of the individuals before losing their earnings and their household circumstances.

Table 7 shows the poverty rates, for different groups of the population before the onset of the COVID‐19 pandemic and after the shutdown considering the policies introduced by the governments. The poverty line is kept constant at 60 percent of the median equivalized household net income in the baseline scenario before the pandemic.

**TABLE 7:** Poverty Rates Before and After the Onset of the COVID‐19 Pandemic

	Individuals Affected by the Shock		Individuals Affected by the Shock in One Earner Family	
Country	Pre‐COVID‐19	Post‐COVID‐19	Pre‐COVID‐19	Post‐COVID‐19
BE	4.73	9.68	11.30	26.70
	(0.516)	(0.893)	(1.439)	(2.419)
ES	16.24	21.68	21.98	37.13
	(0.993)	(1.196)	(2.194)	(2.343)
IT	13.64	30.91	23.08	49.75
	(0.585)	(0.832)	(1.139)	(1.230)
UK	9.10	18.15	26.01	40.16
	(0.424)	(0.662)	(1.164)	(1.257)
	All individuals		Children	
Country	Pre‐COVID‐19	Post‐COVID‐19	Pre‐COVID‐19	Post‐COVID‐19
BE	12.61	13.78	12.34	14.16
	(0.604)	(0.641)	(1.228)	(1.306)
ES	21.06	22.17	26.31	28.05
	(0.596)	(0.609)	(1.137)	(1.142)
IT	20.06	23.57	26.13	32.55
	(0.442)	(0.466)	(0.931)	(0.984)
UK	16.46	18.78	21.39	24.48
	(0.367)	(0.392)	(0.738)	(0.749)

Notes:

Poverty rates based on equivalized household disposable income. The poverty threshold is fixed at 60 percent of the baseline (pre‐COVID‐19) median equivalized household disposable income. Bootstrapped standard errors after 200 replications are shown in parenthesis.

In all four countries, living standards deteriorated due to the COVID‐19 crisis with a large number of workers affected by the labor market shocks falling into poverty. Relative to the overall population, children were also adversely affected by the crisis.

Focussing on the workers affected by the shock, the share of those in poverty before the shock was hugely differentiated across countries, from Belgium characterized by a very low level of in‐work poverty with less than 5 percent of workers in poverty to Spain with a poverty rate of more than 16 percent. The impact of the crisis was disruptive in Italy where the poverty rate, already as high as 14 percent, increased to 31 percent showing the incapacity of the Italian welfare system to offer a good level of absolute resilience. In Belgium and the UK the poverty risk doubled, from 5 percent to 10 percent and from 9 percent to 18 percent, respectively. In Spain the poverty rate increased by a third, from 17 percent to 22 percent.

Individuals living in one‐earner families were, as expected, more exposed to poverty risk relative to all individuals affected by the shock: almost half of them were in poverty in Italy after the shock while in Spain and the UK poverty rates were as high as 37 percent and 40 percent, respectively. In Belgium, where only 11 percent of working individuals in one‐earner families were poor already before the COVID‐19 pandemic, 27 percent were below the poverty threshold after the shutdown.

When extending the analysis to the overall population, the substantial impact of the pandemic on the poverty rate is evident in Italy with a poverty increase of more than 3 percentage points, followed by the UK (2 ppt), Belgium and Spain (1 ppt). The lack of absolute resilience in Italy can be also seen by looking at children who faced a poverty rate after the shutdown as high as 33 percent.

Estimates based on real time data as those reported by Menta (2021) confirm the increase in poverty rates at the beginning of the crisis up to May 2020, in particular with young individuals, women and individuals affected by the shock being the most affected.

## 6 Conclusions

We analyze the extent to which the tax‐benefit systems and earnings compensation schemes in four large European countries, severely hit by the COVID‐19 crisis, provided income support to those affected by the economic shutdown at the beginning of the pandemic (i.e. April 2020). We assess the level of relative and absolute welfare resilience of household incomes during the crisis in Belgium, Italy, Spain and the UK, by simulating counterfactual scenarios with EUROMOD, the European tax‐benefit microsimulation model, combined with COVID‐19‐related household surveys and timely labor market data.

We estimate that on average household equivalized original income dropped substantially by 16 percent–18 percent in Spain and Belgium and as much as 24 percent–26 percent in the UK and Italy. The governments’ fiscal response to COVID‐19 lessened these shocks, leading to smaller average losses in household disposable income of around 4 percent in Belgium and Spain, 6 percent in Italy and 8 percent in the UK. While the overall level of income inequality remained broadly the same, in terms of absolute resilience, the welfare states did not appear sufficiently well equipped to avoid large increases in income poverty.

The differences in the impact of policies across countries arise from four main sources: (1) the asymmetric dimension of the shock by country, (2) the different protection offered by each tax‐benefit system, (3) the diverse design of discretionary measures and (4) the differences in the household level circumstances and living arrangements of individuals at risk of income loss in each country. In particular, earnings compensation schemes provided much needed income protection for households and were the key source of relative resilience in all four countries. Means‐tested benefits in Belgium and the UK and unemployment benefits in Spain also played an important role in protecting incomes, especially at the bottom of the distribution.

Furthermore, our analysis demonstrates on the one hand the cushioning role played by the tax‐benefit system and on the other hand the importance of the income of other household members in providing economic resilience to those affected by the shutdown. The sharing of risks within the household can be seen as an important complement to the insurance function of the welfare state. However, as it is usual in distributive analysis, we have assumed complete income pooling within the household. The possibility that incomes are not in fact pooled serves to remind us of the non‐equivalence of income received in the form of earnings compensation schemes as an individual entitlement on the one hand, and income support schemes, usually assessed on the economic situation of the family as a whole, on the other.

Although we abstract from macroeconomic adjustments and potential behavioral reactions of households to policies, this paper provides a useful methodological benchmark and reference point by which one can evaluate the economic unfolding of the ongoing situation and the new policies that followed those implemented at the onset of the crisis. As mentioned by Clark *et al*. (2020) it is important to understand the mechanisms behind the movement of inequality across countries to disentangle the contributions of earnings shocks and policy responses. Furthermore, the analysis could be extended to nowcast the long‐term (annual) income distribution (Navicke *et al*., 2014) and to consider the impact on material deprivation indicators (Figari, 2012). This kind of analysis would deserve much attention, but it is out of scope of this paper as it requires data not yet available in a cross‐country perspective.

Moreover, our analysis entails the potential economic effects of the first month of the COVID‐19 pandemic and examines the extent of the intended effects of the schemes, though in reality the transfer payments (i.e. earnings compensation and social assistance schemes) were inevitably delayed and this lag might have constrained the liquidity of families with effects on consumption and material deprivation. Consequently, the overall effects of the crisis would be exacerbated if governments do not provide immediately an income stabilization for those experiencing earnings loss, which can potentially translate into further detrimental effects on aggregated demand.

It is clear that the effects of the COVID‐19 pandemic are asymmetric and particularly relevant from an economic perspective for some families and less for others, despite the compensation measures implemented by the governments. It is crucial to take into account such unequal distribution of the shock as the economic consequences are expected to last long and to assess whether the welfare systems are ready for the challenges they have to face (Sacchi, 2018).

Several important policy issues can be highlighted. First, in Italy and Spain, for example, the most important income support schemes depend on past year’s incomes and do not react to a sudden loss of earnings such as those experienced in April 2020. Second, some of the welfare tools deployed during the onset of the crisis do not seem to be well‐thought in terms of design as they provide either lump‐sum transfers or minimum amounts to all those entitled while ignoring previous contribution bases or declared incomes, creating horizontal equity issues. Third, the earnings compensation schemes are capped at a different maximum level across countries which does not resemble differences in earnings distribution or price levels. Last, but not least, some schemes are designed in a way that offers categorical support and prevents full coverage, with domestic workers and several categories of temporary workers being excluded from social protection.

These issues confirm that high levels of efficiency and effectiveness of social protection is key for the sustainability of European welfare systems to allow countries to have effective automatic stabilizers to support incomes during crisis and enable governments to focus on the actions needed for the medium‐ and long‐term economic recovery.

In a cross‐country perspective, the empirical evidence on how well‐suited existing institutional arrangements are for compensating income loss during the pandemic raises normative issues on the protection level that the tax‐benefit systems should guarantee to the population and backs up several longstanding ideas debated in the recent past, such as a Basic Income and a European unemployment benefit. Unconditional Basic Income could make comprehensive compensation possible during the pandemic, without the need of discretionary and temporary policies (Atkinson, 2015). A European unemployment benefit scheme could provide a macroeconomic stabilization and fiscal risk sharing mechanism with interregional smoothing potential as important as intertemporal smoothing potential through debt (Dolls *et al*., 2018). Both ideas, although likely to be developed as academic reflections rather than policy suggestions, can contribute to understand how to cushion asymmetric shocks and provide income insurance to the most vulnerable households in a systematic way, h ighlighting the potential social dimension of the European institutions already reinforced by the common European response to the COVID‐19 crisis.

## Footnotes

^1^
See Eurostat’s indicator “GDP and main components (output, expenditure and income) [NAMQ_10_GDP].”

^2^
Decree Law of the Minister of Economic Development which updates the DPCM March 22, 2020 available here https://www.gazzettaufficiale.it/eli/id/2020/03/26/20A01877/sg.

^3^
Analyzing changes in household incomes in April–May 2020 in the UK, Brewer and Tasseva (2021) find that the Self‐Employment Income Support Scheme has a limited positive effect on household incomes, of 1.3 percent on average.
